# Medical Education

**Published:** 1867-02

**Authors:** 


### MEDICAL EDUCATION.

We have read, with much interest, several articles in our exchanges on this hackneyed subject. Its importance is sufficient to engage, long in the future, the most serious attention, not only of the teachers in our medical colleges and the editors of our journals, but also of every individual practitioner.

One would imagine that the thought of the responsibility, which a young man assumes the day he determines to devote himself to the search for the means of prolonging human life and of relieving human suffering, would be sufficient to stimulate his energies in the pursuit of the knowledge requisite for so high a calling. But the temptation to avoid severe mental labor and the want of due appreciation of the duties of a physician are so great, that thousands who commence the study of medicine, and thousands who continue in its practice, apparently strive to prove how little they can learn.

The obstacles which tend to prevent diffusion of thorough medical knowledge in the profession are, principally:—1st. The difficulty of so far overcoming the ignorance and prejudices of the masses that they can recognize the merits of well-educated physicians, as compared with the pretensions of charlatans.

2d. The imperfect preliminary education of a large proportion of those who enter upon the study of medical science.

3d. The want of suitable legislation in reference to several important points connected with the practice of medicine.

4th. The imperfect system of instruction in our medical colleges.

These obstacles will, undoubtedly, to a certain extent, be finally overcome. They are all, however, so intimately related with each other, and are so closely dependent upon the character of our national habits and the spirit of our institutions, that change for the better must, necessarily, take place very slowly. It would be difficult to state just where the chief fault exists. We are confident it does not rest with our medical colleges alone.

We believe these institutions, in view of the manner in which nearly all institutions of learning in this country are developed and supported, and in view of the difficulties they encounter, accomplish, upon the whole, as much for the educational interests of the profession as can reasonably be expected. The average standard of mental capacity and acquirements of the members of our profession engaged in public instruction, is certainly much above that of those not thus engaged. The duties of our medical teachers, as a class, are faithfully discharged.

We believe, moreover, nearly all our medical colleges, however imperfect the result of their labors may now be, will become institutions of which our profession may well be proud.

It should be remembered that medical colleges in this country are, with few exceptions, private institutions, dependent upon private capital for their origin, and dependent for their support upon the fees received from students. In almost all respects, our colleges are fully equal to the demands of the class of students who attend them.

It is doubtful whether, at this day, any of our medical colleges, thus dependent upon private capital, would secure the attendance of a sufficient number of students to keep it in existence, if every pupil was excluded who was found, after a fair examination, absolutely deficient in his preliminary education, and who could graduate only on passing a reasonably rigid examination by a board of competent physicians not connected with the college.

The courses of instruction are as long and as thorough as the resources of the majority of students will admit. A large proportion of them are unable to attend "summer courses," but are compelled to pursue their studies during the interval between the courses of lectures, under the direction of physicians in active practice. They have an opportunity of observing a large number of cases under the charge of their instructors, and are often called to assist in surgical operations, in dressing wounds, in applying bandages and splints, and in attending and nursing the sick. Under the guidance of their preceptors, they gain a knowledge of auscultation and percussion, and the other modes of diagnosis. Students who thus spend eight months of the year in acquiring a knowledge of the "practical parts" of their profession, are, really, receiving clinical instruction.

From quite extensive acquaintance with students of medicine, we are led to believe they are disposed to devote their time to the "practical parts" of medical science, to the neglect of the so-called "theoretical parts"—of physiology, histology, and pathology. For students who have been eight months in the year receiving clinical instruction, under the direction of pre ceptors, we believe the advantages of clinical lectures during their first two years in college are much overrated. The welfare of such students really requires that their attention, the first two courses in college, should be especially directed to those branches which are too often regarded as "unpractical."

If they attend regularly the lectures on anatomy, physiology, chemistry, and materia medica, and spend sufficient time in the dissecting room to learn, by careful observation, the anatomical character and relations of every tissue and organ, and, with this labor, read appropriate works on these subjects, they have little time for clinics at the hospital.

It is unnecessary to dwell upon the importance of a good preliminary education for young men about to enter upon the study of medicine, and yet it is difficult to say how much or how little knowledge of the languages and of the sciences are requisite. Our colleges are, undoubtedly, slowly raising the standard of scholarship of the classes which take the degree of doctor of medicine. We do not believe, however, any radical improvement in the system of medical instruction, as regards the preliminary education of the students admitted into the schools, the mode of teaching, or the examinations for a degree, will take place, until the people of this country take the same interest in the medical colleges which they have in our literary colleges and in our scientific and theological schools. Would not well-directed efforts on the part of our colleges, of our medical journals, and of the profession generally, turn the attention of the public and of our wealthy citizens to the importance of encouraging thorough medical education.

While most noble bequests and donations have been made to other institutions of learning and to hospitals, comparatively little has been given to medical colleges. When we consider the important duties performed by the medical profession, in every civilized country, and how dependent upon the knowledge and skill of physicians mankind is for the relief of suffering, for the preservation of the public health, and the prolongation of life, it is scarcely explicable why benevolent men, of unbounded wealth have not more largely contributed to the establishment and support of colleges for the education of physicians and surgeons. We hope the time will come, when some generous man, of princely fortune, mindful of the unspeakable blessings which medical science can confer upon mankind, will supply the means of constructing and endowing a medical college equal to the actual wants of the present time. We can readily conceive of such a college—a noble edifice, as beautiful in structure as it is well adapted for its purpose; its lecture-rooms large and attractive; its museum rich in everything that can illustrate the condition of each organ, in health or disease, and containing specimens of every article in the materia medica; its laboratory supplied with ample apparatus for teaching chemistry; its library filled with valuable works in every department of medical science; and its extensive dissecting room supplied with every convenience for teaching practical anatomy and operative surgery.

A sum of money equal to a-third of a single bequest which has been made in this country for a noble charity, would be sufficient to establish such a college and endow every professorship.

The course of instruction should continue through a period of three, if not four, years, each year being divided into two terms of twenty weeks each. The mode of instruction, especially during the first two years, should be almost entirely confined to the systematic study of suitable text-books and *recitations*, precisely as in our literary colleges. There is nothing, either in the kinds of knowledge sought by the students in our colleges, or in the mental capacity and training of these students, which renders it more advantageous for them to receive, passively, instructions by lectures, rather than to be obliged to study stated lessons till they are able to recite them. There should be but three or four such exercises each day, and, possibly, in winter but two, thus giving the members of the class ample opportunity to dissect. Anatomy, physiology, and chemistry are sufficient to occupy the undivided attention of the class during the first year.

There are excellent text-books in each of the other branches of medicine, small enough, and yet sufficiently comprehensive for beginners, which could be carefully studied and recited through during the second year. The third year would then remain for a general review of each subject, and for clinical instruction. Students could be admitted to the classes of any term, provided they passed a satisfactory examination in the studies pursued during the previous terms.

The introduction of such a plan of education in our colleges, with the means now at their control, must be considered, practically, as an impossibility. But in an institution amply endowed, as we have supposed, a sufficiently large faculty of learned physicians, experienced in lecturing and clinical teaching, could be supported with but a small fee from each student in addition to the endowments of the professorships. The recitations could be conducted by teachers corresponding to the tutors of our literary colleges. While the number of medical men who are able to instruct students in all respects acceptably by lectures is comparatively small, there are in every city many young practitioners who have, in their early life, enjoyed considerable experience in teaching, and who are eminently capable of conducting classes in the recitation room, through our best medical text-books. There are many such young men, who could not only teach most acceptably, elucidating obscure points and explaining the relation of important facts in the different branches of medicine, but who, for the purpose of training themselves for medical teaching and for general self-improvement, would willingly, for a comparatively small fee from each student, devote an hour daily in performing the duties of medical "tutor."

The advantages of this mode of instruction are apparent. The student is obliged to study carefully and impress upon his mind the subjects he is learning, in order to appear creditably in the recitation room. He acquires the habit of expressing his ideas definitely and in scientific language in the very act of reciting. It is also incalculably more advantageous to the student to be compelled to perform with his own hands, under the guidance of his tutor, the various operations required in practice, than simply *witnessing* their performance. Every student should, therefore, be obliged to spend an hour a day, for a sufficient period, in applying the various kinds of splints and bandages to different portions, either of suitable models or of the human subject; he should practice his sense of touch and hearing in palpation, auscultation, and percussion of different portions of the healthy body; he should frequently inspect the throat and ears, for instance, of individuals in health, and in all these exercises he should always be required to describe, minutely, each portion of the organs examined. Each student should have the opportunity of applying obstetrical instruments upon suitable manikins of the pelvis and fœtus. When possible, the instructor should remove the pelvic viscera from the female cadaver, and place within the pelvis a fœtus, in different positions, for the student to examine per vaginam, and thus educate the finger in detecting the sutures, fontenelles, and other portions of the fœtus. In this way, the operations of turning and of applying the forceps can be repeatedly performed by the student, who can thus, most certainly, better prepare himself for the performance of these operations in actual practice, than by simply listening to descriptive lectures. Whatever objections there may be to this mode of teaching medical science, we believe the greatest good to the greatest number of students would be ensured by its general adoption.

We have thus far regarded the labors of the student, simply as they are preparatory to the study of disease. These preparatory studies are sufficient to enlist his whole energies, at least one or two years. As it is the great object of these studies to fit the student for the acquisition of a knowledge of everything connected with morbid phenomena and of the effects of remedies, every qualified student and practitioner should have access to the wards of a hospital.

The bedside offers, perhaps, the most important field of labor for the medical teacher. Even now, the reputation of medical colleges depends, in a very great measure, upon the facilities the students enjoy for clinical observation. We believe, however, that the present mode of instruction at the bedside should be somewhat modified. The clinics should be given daily, and the number of students admitted to any ward at one time should be limited.

Increased facilities for small classes to secure private clinical instruction from the resident physicians of our hospitals would be desirable. In this way, advanced students and practitioners desirous of reviewing certain studies, could secure more satisfactory opportunities of practicing auscultation and percussion, and of examining at leisure cases of syphilis, diseases of the eye, skin, and special organs.

Utopian as may seem the plans of medical instruction above delineated, we believe the time will come when the means necessary to render their adoption practicable will be provided, either by liberal bequests and donations from our wealthy men, or by grants from the State.

